# Epidemic Cholera

**Published:** 1847-12

**Authors:** 


3. *Epidemic Cholera.*—This epidemic is steadily advancing westward, by the same route which it pursued in 1831, and at the last accounts had reached Central and Southern Russia. Without counting Georgia, Caucasus, and the country of the Cossacks of the Black Sea, it already reigns in sixteen governments. On the 30th of October it broke out at Moscow. The disease has also broken out anew in Persia. Three cases are said to have occurred at Paris; two of these did not present any very characteristic symptoms, but in the third there were cyanosis, rice-water dejections, &c.—*Ibid.*

